# Saturn's Kingdom; or, Fable and Fact

**Published:** 1892-03

**Authors:** 


*Saturn's Kingdom; or, Fable and Fact.* By CHARLES MOORE JESSOP. Pp. xiii., 294. London: Kegan Paul, Trench, Trübner & Co. 1891.

This book might well be entitled the evolution of

"The great globe itself [and] all which it inherit."

It begins with the mythological story of Saturn, as the son of Heaven, because the apparent motion of the starry universe is a measure of time; and as the son of Earth, because the ebbing and flowing of the sea is likewise a measure of time; and continues with the mythical Bathybius of Hæckel, and the story of the Neanderthal skull and other beginnings of man himself. It then leads up by numerous entertaining chapters on a multiplicity of subjects to the time when "life on earth may have terminated from a deficient food-supply for increased population; or from the decrease of intrinsic and extrinsic heat; or, through the onward course of the solar system, this earth may have come into contact with some dark body of another system of equal magnitude moving at an equal rate, when collision would at once terminate its career." It is obvious that the book is crowded with topics of surpassing interest.

